# Integrating biomedical and herbal medicine in Ghana – experiences from the Kumasi South Hospital: a qualitative study

**DOI:** 10.1186/s12906-016-1163-4

**Published:** 2016-07-07

**Authors:** Millicent Addai Boateng, Anthony Danso-Appiah, Bernard Kofi Turkson, Britt Pinkowski Tersbøl

**Affiliations:** Global Health, Department of Public Health, CSS Øster Farimagsgade 5, DK-1014 Copenhagen, Denmark; School of Public Health, University of Ghana, Accra, Ghana; South Suntreso Hospital, Kumasi, Ghana

**Keywords:** Herbal medicine, Biomedicine, Integration, Qualitative research, Ghana

## Abstract

**Background:**

Over the past decade there has been growing interest in the use of herbal medicine both in developed and developing countries. Given the high proportion of patients using herbal medicine in Ghana, some health facilities have initiated implementation of herbal medicine as a component of their healthcare delivery. However, the extent to which herbal medicine has been integrated in Ghanaian health facilities, how integration is implemented and perceived by different stakeholders has not been documented. The study sought to explore these critical issues at the Kumasi South Hospital (KSH) and outline the challenges and motivations of the integration process.

**Methods:**

Qualitative phenomenological exploratory study design involving fieldwork observations, focus group discussion, in-depth interviews and key informants’ interviews was employed to collect data.

**Results:**

Policies and protocols outlining the definition, process and goals of integration were lacking, with respondents sharing different views about the purpose and value of integration of herbal medicine within public health facilities. Key informants were supportive of the initiative. Whilst biomedical health workers perceived the system to be parallel than integrated, health personnel providing herbal medicine perceived the system as integrated. Most patients were not aware of the herbal clinic in the hospital but those who had utilized services of the herbal clinic viewed the clinic as part of the hospital.

**Conclusions:**

The lack of a regulatory policy and protocol for the integration seemed to have led to the different perception of the integration. Policy and protocol to guide the integration are key recommendations.

## Background

Over the past decade there has been a growing interest in the use of herbal medicine both in developed and developing countries [1]. Estimates reveal that 80 % of patients in Africa use traditional medicine [2]. In Ghana about 70 % of patients are known to use herbal medicine [2, 3]. Herbal medicine is one of the classifications of traditional medicine [4]. Others grouped under traditional medicine include using animal parts and minerals for the treatment or prevention of a disease or ailment [4]. In the African context, traditional medicine comprises knowledge and practices, whether explicable or not, used in the diagnosis, prevention and elimination of physical, mental, or societal imbalance, and relying exclusively on practical experience and observation handed down from generations, verbally or in writing [5]. Herbal medicine is the most widely used among the traditional medicines [6], and differs from region to region, even within a particular country [7]. In South Africa alone, 27 million people are estimated to use traditional medicine for their primary healthcare needs [8]. The preference for herbal medicine may be attributed to the perceived high cost of allopathic medicines, and cultural and spiritual factors [8]. Patient-oriented practices and services by traditional providers may be another reason for patients’ preference for herbal medicines [9].

Views on herbal medicine use in Africa differ among biomedical health professionals. A study from Nigeria showed that 41 % of physicians interviewed believed that herbal medicine could be effective for some of the chronic diseases [10] although most still thought herbal medicine could only be effective if considered as a complementary medicine [10]. A study conducted in two provinces in South Africa suggested that although herbalists were willing to work with biomedical practitioners, the collaborative effort was not equally received by biomedical practitioners [11]. The study also showed that although some community health workers had personal experience with traditional or herbal medicine, they were less likely to recommend it to patients [11, 12]. The process of integrating herbal medicine with biomedicine has been slow and difficult in most countries [12, 13]. In Ghana, anti-malaria herbal medicines are often used to complement allopathic anti-malaria drugs [14]. A recent study on medicines use by pregnant women visiting health facilities showed that regardless of the prescription given, most women with malaria also used some herbal medicines [15], even when some of these medicines had clear caution against use by pregnant and lactating women [15].

In Ghana there are currently six known biomedical research institutions of which four mainly conduct testing for safety and efficacy and two for microbiological testing of herbal medicines [16]. The six institutions are Noguchi Memorial Institute for Medical Research; Centre for Clinical and Pharmacology and Therapeutics, University of Ghana (UG); Faculty of Pharmacy, Kwame Nkrumah University of Science and Technology (KNUST); Centre for Scientific Research into Plant Medicine (CSRPM) [17] and the Department of Chemistry, University of Cape Coast (UCC) [16]. Whilst the Noguchi Memorial Institute for Medical Research is responsible for conducting both microbiological and biological testing [16], the Centre for Scientific Research into Plant Medicine (CSRPM) combines research, production of herbal medicine and clinical care [17] and KNUST trains herbal medical practitioners similar to the training provided for biomedical practitioners in Ghana [16, 18].

Bodeker discusses two models of integration [12], from the developing countries experience, namely 1) integration of biomedical and traditional medicines through medical education and practice, and 2) parallel integration. Integration through medical education recognizes and incorporates traditional medicine in the health care delivery system and also covered by national health insurance [19]. China is an example of this model as the country has successfully integrated traditional medicine into its biomedical health system [12, 13]. For instance, 95 % of hospitals have traditional medicine departments and these departments serve 20 % of in-patients on a daily basis [12]. Parallel integration involves separation of biomedical and traditional medicine in the national health system [12, 19]. This model is commonly found in African countries like Nigeria, Guinea and Ghana but also some Asian countries such as India and South Korea [19] This model does not usually involve formal educational training or utilization of the formal health care delivery system and thus regulation [19]. It may also not be supported by health insurance systems. The WHO adds a third model namely, Tolerance Model where biomedical health facilities are encouraged to compliment delivery of care by some traditional medicines which have been endorsed by the country’s legislation [19].

Hierarchical medical pluralism within a nation is a universal phenomenon and has been discussed in terms of the analytical concepts of structural superiority (the power, prestige and wealth the system is granted) and functional strength (how widely the system is distributed and utilized) [13]. A medical system (e.g. biomedical health care) is structurally superior when it has a high level of power, prestige and wealth and functionally strong when it is widely utilized by the population. Different medical systems are most often in competition with each other and the hierarchical nature of relationship may lead structurally superior systems to dominate other types of health care [13]. We used this differentiation in our study to help describe the position of herbal medical services in relationship to biomedical services in the process of integration at Kumasi South Hospital.

Foucault’s work on governmentality and health is concerned with power relations in medical systems. Foucault explains bio-power as the situation where the population and their welfare becomes more structured and organized for the purpose of increasing productivity in the society or country [20]. An aspect of this is disciplinary power, which has the basic aim of creating compliant human beings [20]. Governmentality is viewed as a major influence on the practices of the people being governed and Foucault explained this as governing the people in terms of their social and cultural conduct rather than the administrative structures of the people [21]. This concept of governmentality and bio power are useful for analysing and discussing the form of integration practiced and how it is perceived by stakeholders.

### Rationale

In April 2001, a council was established to regulate the practice of herbal medicine in Ghana. The recommendations of the Council allowed hospitals and pharmacies to prescribe and dispense certified herbal medicines [12]. Then, a policy on the practice of herbal medicine was established in 2005 followed by a recommendation on integration of biomedicine with herbal medicine and this has been piloted in some hospitals since 2011 [16]. For the past years, there has been no document or study on the progress and perception of users and health practitioners on this integration. This study explored integration of herbal and biomedical health services within a Ghanaian health facility by investigating the practices and perception of patients, health workers and selected key informants.

## Methods

### Study design

This study used a qualitative phenomenological research design to collect data and explore the integration of herbal medicine with biomedicine in a hospital in Ghana. In phenomenology, the researchers work to develop a solid pre-understanding, based on existing literature, and critical reflection on the research topic, aided by critical questioning of the pre-understanding by peers and colleagues. Pre-understanding of the phenomenon and existing literature [21, 22], helped to develop the research tools. The researchers resorted to the skills of probing further on the responses provided by respondents in order to better understand their personal experiences and not as preconceived. The study investigated experiences and perception of stakeholders described below, in April 2014. A purposeful and snowball sampling methods were also used to help achieve maximum variation in the study sample.

### Study location

The study took place at the Kumasi South Hospital and settings of the five key informants mentioned below. Kumasi South Hospital is the second largest hospital in the southern part of the Ashanti region of Ghana and it is one of the 17 hospitals in the country where integration of herbal medicine introduced by the Ministry of Health was piloted. It is located within the Kumasi Metropolis with a population of 582,877 and renders services such as general medical care, public health, diagnostics, training of medical residents and specialist services including the herbal clinic.

### Study population

A total of 34 participants took part in the study of which five were key informants from Faculty of Pharmacy (FOP), the institution that trains medical herbalist at the Kwame Nkrumah University of Science & Technology [1]; the Pharmaceutical Directorate of the Ministry of Health [1]; the Medical Herbalist Association [1]; the Ghana Federation of Traditional Medicine Practitioners [1] and the hospital management at Kumasi South Hospital [1]. These interviews focused on background insight from the perspective of trainers and policy makers about initiation of the integration. The other 29 participants included 10 patients from the biomedical unit; 6 patients from the herbal clinic; 2 biomedical practitioners; 2 medical herbalists (one of whom was the manager of the herbal clinic); 8 nurses from the biomedical unit and 1 nurse from the herbal clinic. All participants were selected purposefully based on the relevance of their experience to the study objective.

#### Data collection

The data were collected through key informant interviews, in-depth interviews, focus group discussions and participant observations. One focus group discussion involving 5 nurses was held at the biomedical unit of the Kumasi South hospital. The questions in the in-depth interviews and focus group discussions were the same. Participant observation was carried out on the compound of the hospital between the herbal clinic, the reception and the waiting areas at the biomedical out patients’ department (OPD) and was focused on how patients entered the facility and how choices were made about type of health care services to utilize. Some patients were interviewed at the OPD but most were interviewed at the hospital pharmacy during the final stage of their visit to the hospital. The question guides for interviews and focus group discussions were semi-structured and aimed to gain an understanding of the perception of study participants concerning the integration process. Participants of this study were assured of anonymity and confidentiality. All respondents were either interviewed using pre-tested questionnaire in English or the local dialect Twi. The language used in each interview depended on what each study participant was most comfortable with.

### Data management and analysis

Based on Giorgi’s phenomenological framework [23], interviews collected were transcribed and read through thoroughly. In this study, transcription was done twice (first by the research assistants and the second by the lead researcher) as a quality check. Analysis of data for phenomenological studies does not only include the responses provided by participants by word of mouth, but also, the tone of speech, body language and even silence are considered in analysing the data [24]. Responses were captured in notes during interviews to enable the researchers to better understand and analyze the perception of the respondents. The responses were coded and recoded before grouping into themes, summarized and further analysed. Insight gained from interviews, focus group discussions and observations was compared to triangulate the responses to ensure validity of the data.

## Results

Demographically, patients interviewed in this study had an age range between 29–80 years and they included 3 men and 13 women. Two (2) of the male patients were from the biomedical treatment section of the hospital and the other from the herbal clinic. The majority (7 out of 13) of the female patients were from the biomedical treatment section of the hospital as well. Cases presented by patients from the biomedical unit included eye problems, diabetes, hypertension, pregnancy and pains in the knees. The patients from the herbal clinic commonly reported cases of general pains in the stomach, legs and waist with some cases of hypertension. Although none reported malaria, most of them said they would utilize the herbal services for treatment of malaria.

The total number of health workers interviewed was 12: biomedical practitioners (2), Medical Herbalist (2) and Nurses (8). Of these, seven were from the biomedical section and one from the herbal section. The length of time these health workers had been at post varied from one month to 28 years, with most of the Nurse respondents being general Nurses. The biomedical practitioners were both interviewed individually in one of their consulting rooms and both were female practitioners with five and seven years of working experience. The medical herbalist and the general nurse at the herbal clinic were interviewed in the consulting room at the herbal clinic. The study showed that in Kumasi South hospital the biomedical unit and herbal clinic are located on the same compound and managed by one common administrative unit, headed by a biomedical superintendent. The herbal clinic is located about 50 m from the main entrance of the hospital. Its greenish look and the inscription ‘herbal clinic’ distinguishes it from the main hospital block housing the biomedical unit. From participants’ observation, almost all the patients went straight to the biomedical unit located at about 60 m from the entrance. The study showed that patients could access herbal medicine treatment in three main ways. The first was at the OPD of the biomedical unit where patients got their folders and checked their vital signs. This is the point where patients were separated to go to either biomedical or herbal unit. While biomedical patients received their folders, checked their vital signs and waited for their turn at the OPD, patients who wanted to use the herbal clinic were directed there after picking up their folders. These patients had their vital signs checked at the herbal clinic. Patients who attended the herbal clinic had their folders kept there for their subsequent visits. The second way a patient could access herbal medicine treatment was when the biomedical practitioner recommended herbal medicine as an alternative treatment. In those cases, the biomedical practitioner informed the medical herbalist directly, prior to the patient’s visit. The third way was self-referral of the patients themselves to the herbal clinic. In this case, the patient would acquire a new folder which would be kept at the herbal clinic for their subsequent visits.

With regard to when the integration process was initiated in Ghana, we obtained mixed answers. Whilst some key informants asserted that it was initiated from a WHO recommendation that asked countries to integrate biomedicine with traditional medicines into their healthcare delivery systems others indicated that the first graduates from the Herbal Medicine Department of the Faculty of Pharmacy, KNUST, initiated the process of the integration when they sent a formal proposal to the Ghana Ministry of Health. This view was shared by the Medical Herbalists Association as well as GHAFTRAM. According to them, the proposal was sent in 2011 and the integration process was initiated the same year. Although all key informants would associate the start of the integration to the WHO policy on traditional medicine or the proposal from the medical herbalists, no one could give a detailed description of the steps of the decision making process leading up to the pilot stage of the integration. The study found that 17 public health facilities across the country including Kumasi South Hospital had piloted the integration since 2011.

Efforts were made to locate or identify policy documents, guidelines or standing operating procedures governing the integration practices. However, we were unable to identify such documents. The only policy document some key informants referred to was the Traditional Medicine Practitioners Act 2000, which sought to regulate traditional medicine use in the country. However, we did not obtain any reliable policy information on the integration.

The study showed that medical herbalists were familiar with the Traditional Medicines’ Act, but not of any policy on integration. They were of the view, however, that the existence of such a policy would be useful for the sustainability of the integration. Key findings and quotes from study participants have been assembled in a table to aid an easy overview of the different perception and experiences.

The content of Table 1 underlines the variation in responses by the different study participants on how they viewed the phenomenon of integration of herbal medicine with biomedical services. The study showed that key informants were supportive of the initiative but acknowledged that the system had not fully integrated. While biomedical health workers perceived the system to be parallel than integrated, herbal medicine health workers perceived the system to be more integrated. Surprisingly, patients at the biomedical unit were not aware of herbal clinic in the hospital. Patients who had been to the herbal clinic viewed it as part of the hospital’s formal services. The findings also showed that there was no policy to regulate the process of integration. The responses in this table were collected with the aid of some interview guide.

**Table 1:** Perceptions by themes and respondents The content of the table underlines the great variety in how different study participants see the phenomenon of integration of herbal medicine with biomedical services

Patients	Health workers			Key informants
	Biomedical Nurses	Biomedical doctors	Medical herbalist	
Theme: perceptions of herbal medicine:				
Patients generally used herbal medicine alongside biomedicine for various reasons: -“*some diseases do require herbal medicine or a combination of both” (female, food seller, 41 year)* *- when biomedicine is not effective enough or does not entirely solve the problem, herbal medicine may be used” (P5, female, dressmaker. 29 years)* Patients also mentioned concerns about herbal medicine in general: - “*they are good especially when you get good herbal medicines with the correct prescription. The fear is that most of them are without prescription, sometimes bad people also prepare unsafe herbal medicines” (P5, female, dressmaker. 29 years)* - “*the dosage is not always accurately dispensed*” (P1, male, retired chief inspector, 72) Patient mentioned their interest in seeing biomedical technology to the practice of herbal medicine: *“if you can use machines to detect the level of cure or treatment of diseases with the herbal clinic, we would appreciate it” (P1, male, retired chief inspector, 72 years)* One patient was an herbal medicine provider and said: “*Yes, I am an ardent user of herbal medicine. God gives me visions and I give them certain medicines to treat their illness. Some patients came to me suffering from fibroid and I gave them herbal medicines for treatment. I personally use it too and because of my constant usage I don’t fall sick a lot”(P2, female, farmer, old woman not certain of her age)*	The majority of the nurses acknowledged the important role that herbal medicine plays in patients’ treatment choice The providers also shared their own interest in using herbal medicine. A staff nurse said: “*Herbal medicines are good. I used herbal medicines when I was not [yet] a health professional. I will recommend herbal medicines to other patients*” The tendency of some herbal medicine practitioners to claim that one remedy could cure several illnesses was found to be dishonest Nurses applauded that scientific methods were increasing applied in testing and prescriptions of herbal remedies. It was noted that it is better that herbal remedies be prescribed within the hospital setting than outside: *“Herbal medicine in the hospital now goes through research before it is administered. Also people now accept it because it is in the hospital and prepared under hygienic conditions” (female nurse, interview)* *“It motivates patients to come to the hospital because herbal medicine is easily accessible” (male, anesthetist, FGD).*	Doctors were generally skeptical towards herbal medicine but thought it safer when provided from the hospital *I think it would be better to come to the herbal clinic for medicines than go to any place for medicines” (female, doctor 1)* *“I sometimes refer patients to herbal clinic especially patients who are not financially sound to purchase their drug, provided there is an herbal medicine option” (female, biomedical doctor 2).* “*There have been proliferation of herbal medicines and structures have been built for that purpose. People are trained to cater the needs of patients. I have used herbal medicines for food poisoning*.	The medical herbalists all appeared enthusiastic about Positive towards the concept *“We respect each other but how integrated the system is, is dependent on individual’s perspective.”* *(Medical herbalist, 2.)* *“When patient’s condition requires surgical operation, the case is referred to the main hospital.* *(medical herbalist 1)*	Key informants all expressed positive sentiments about herbal medicine in the sense that they supported the integration in theory: *“Ghana has started some kind of integration of herbal treatments into the health system. Currently, it is parallel”. (Ministry of Health)* *“The system has not been fully integrated”. There is sometimes cross referral”.* *(hospital management)* *“Sometimes, we become confused with collaboration and integration. It is not fully integrated” (GHAFTRAM).*
Theme: Perception of integration				
Patients we encountered in the out-patients’ department were not always aware that there was a herbal clinic on the compound Some patient saw the integration as a way of authorizing the use of herbal medicine. *“As you can see, this means herbal medicines are good. Some diseases require the use of herbal medicines so people will come here for treatment.” (P4, female, 41 year, biomedical)* *“Not aware of existence of herbal clinic. Some disease require herbal medicine” (P2, old female, not sure of age, biomedical)* Patients were positive towards the concept, they felt it was safer. “*It is a good initiative. The herbal medicines will complement the biomedical medicines” (P6, male, 36 years, biomedical)* “*It very good. because when visited, the main hospital and herbal clinic and combined, both really worked well”* *(P16, male, 51 year; P20, female 60 year, herbal)*	Nurses did not perceive service to be integrated. *“we are not integrated or working together” (female, nurse 1)* *“Not really integrated. It is seen as another unit. It is the patient's choice” (female, nurse, FGD)*	Did not see integration but parallel collaboration *“not for see any integration anytime soon,* *“It has not really worked, I think it is more parallel” (female biomedical doctor 1)*	Perceived a strong integration *“we work hand in hand with biomedical unit” (medical herbalist 2)*	Positive towards integration *“We decided to assign some general nurses to the herbal clinic when it started with one medical herbalist”. We did this on our own.” (Hospital mgt.)* *“I don’t have a problem with either it being integrated or parallel. The main focus is to make the patient satisfied. The second is to make sure it is of high quality”(association of medical herbalists)*
Theme: Experiences of integration-related practices				
Patients used the same laboratory *“At the herbal clinic, they let you go for laboratory tests before you are treated. I think they help”. (P20, female, 60 year, herbal)* *“I first went to the hospital for my folder because I had used the biomedicine and it did not help me.” (P17, female, 21 year, herbal)*	Some nurses saw the out-patient department as the only point of integration “Patients go straight to the herbal center when they have their folders from the OPD and most people like herbal medicine” (female, nurse, FGD) *“We have not admitted any patient who uses herbal medicines and biomedical medicines. I don’t think it is integrated, because we are not working together with them”. (female, nurse, interview)*	Doctors’ experiences concerning integration-related practices varied greatly. Some did not see any integration: *It has not really worked. You are in between two choices. It is the patients’ choice to choose between biomedical medicines and herbal medicines. It is not really integration.* *(female, senior medical officer)* *“There is no information flow between us. But they sometimes have presentations on herbal medicine in Ghana”. (female, senior medical officer 1)* Another doctor saw it differently: *The information flow is very good. We have the prescribers meeting. They decide what is to be done. They make presentations on what has happened* (female, senior medical officer 2) *Yes, we do transfer some of the patients to the herbal clinic especially the men who suffer from ED. The herbalist is my friend. The biomedical drugs are expensive* *(Female, senior medical officer)*	Medical herbalists perceived the out-patient department and the laboratory as points of integration. *“The integration system is very strong. There are sometimes intra referrals from herbal to biomedical and* vice versa*”.* *“Patient goes to OPD, then to herbal clinic, we check vital stats, then patient will be examined during consultation and referred to the lab if necessary. We ensure continuity for review” (medical herbalist 1).*	Key informants perceived referrals as ways of integration. *“In case there is a challenge, we confer with them. We do cross referrals. If we realize that a patient needs the biomedical medicines, then we refer them to the main stream hospital. On the other hand, we get cases if a patient has been on biomedical medication and still it is not working”. (association of medical herbalist)*

Note: Kindly insert table just before the summary of findings in the results section

## Discussion

The inclusion of herbal medicine at biomedical facilities and piloted at 17 hospitals in Ghana attest to the important role herbal remedies play in the populations’ treatment practices. The absence of a policy that outlines the goals and scope of the integration is interesting and probably also one of the explanations why study participants viewed the integration so differently. It is unknown to all what integration actually meant and the procedures and practices that should be in place for the two kinds of services to be integrated. The phenomenon under study here is thus illusive: study participants had ideas about what constituted integrated services but there were no agreed criteria to determine how and when services could be considered integrated.

In Ghana, the biomedical health care system can be viewed as structurally superior, whilst herbal medicine, and possibly other local healing traditions, can be viewed as functionally strong. Herbal medicine does not have the same privileges in terms of Government support, coverage by national health insurance scheme and infrastructure as the biomedical health care system, although it is widely practiced and utilized by the population. Biomedicine can be perceived to rank higher in the hierarchy of health care systems given its association with modernity, technology and science. For patients and professionals, this association to science and technology is important and may influence their health seeking behaviour. When patients discussed herbal medicine in relation to integration, they stressed the attractiveness of transferal of biomedical practice technology such as research into efficacy and dosage standardization as well as the attractiveness of integrating biomedical procedures such a laboratory tests into herbal medical services. A study from Ghana [25] showed that biomedical practitioners were willing to support the integration if only it was a directive from the Ministry of Health with well outlined roles and operating procedures applying to all stakeholders.

Considering the concept of governmentality - the government of peoples to fulfil the state’s objectives [20] the move to integrate herbal and biomedicine can be seen as an attempt to discipline and control both the user, the provider and the herbal medicine remedies. This is by way of directing the population towards providers using herbal medicine that is closely associated to and governed by scientific scrutiny and standardization: produced, packaged, and dispensed under the control of the health care facility. A study in Vietnam [26] about modernizing herbal medicine through the scientific approach considered this sense of control as a way of protecting and promoting public health [26]. In this process, although the objective is to promote the health and welfare of the population, it is also a way of controlling the use of herbal medicine in the country. As can be seen in this current study, one objective is to provide a one-stop treatment point for patients, which can be explained by the concept of governmentality as creation of a compliant being.

The integration could also be seen as a move to control the practice of herbal medicine, favouring those who have a university degree over those who practice herbal medicines in the communities. As could be seen in the study on “a traditional healers’ training model in rural Nepal, strengthening their roles in community health” [27], the introduction of herbal medicine in Ghana is a way of officially recognizing herbal medicine as a treatment option in the country and as well a way of controlling the practice of herbal medicine in Ghana.

### Limitations

The responses of most respondents may have been influenced by the setting where the study took place because although studies show that 70 % of Ghanaians use herbal medicine [28], the responses from this study showed that most patients would prefer biomedicine to herbal medicine. Also since this hospital is the second largest hospital in the south of Ashanti Region, it is possible that some patients were on referral from other hospitals and this might have accounted for the responses that they did not know the existence of herbal clinic. All the same, since this study adapted phenomenology, which seeks to describe the experiences of people just as they are and not to analyze [21], the study reported the responses exactly as provided. This is likely to introduce some variation in responses from a study which is carried out with the same objectives but different setting. Whilst there may be limitations inherent in the study design and methods used, these by no means compromise the results reported. Being a qualitative study with the small sample size and mostly based on subjective answers limits the extent to which these findings can be generalised le.

## Conclusion

The study shows that integrating herbal medicine into a biomedical health facility or hospital is understood differently by different stakeholders. There were also varied perceptions on the kind of integration operating in the hospital and whilst this could be attributed to the absence of a clearly defined policy, this has serious implications on the success of the integration process. With the study design and a population within a similar context like the hospital chosen, this study could be applied in a mixed sample population (patients, medical herbalists or traditional practitioners, bio-medical practitioners and management) and similar findings might be acquired. This study recommends a larger multi-facility study in representative hospitals in the country which are undergoing similar health system changes to contribute generalizable evidence to inform on the integration process and future policies.

## Abbreviations

GHAFTRAM, Ghana federation of traditional medicine practitioners; OPD, out patient’s department
